# Metabolic Effects of Cellular Necrosis Caused by Exfoliative Toxin C (ExhC) from 

**DOI:** 10.1021/acs.jproteome.4c01029

**Published:** 2025-05-22

**Authors:** Carolina Gismene, Fábio Rogério de Moraes, Anelize Bauermeister, Thyerre Santana Da Costa, Marilia de Freitas Calmon, Luís Eduardo de Almeida Passos Cerbino, Paula Rahal, Rejane Maira Góes, Luiz Alberto Beraldo de Moraes, Ljubica Tasic, Raghuvir Krishnaswamy Arni

**Affiliations:** a Multiuser Center for Biomolecular Innovation, São Paulo State University - UNESP, São José do Rio Preto, SP 15054-000, Brazil; b Department of Chemistry, Institute of Chemistry, University of São Paulo - USP, São Paulo, SP 05508-000, Brazil; c Institute of Chemistry, Universidade Estadual de Campinas - UNICAMP, Campinas, SP 13083-970, Brazil; d Laboratory of Genomic Studies, São Paulo State University - UNESP, São José do Rio Preto, SP 15054-000, Brazil; e Department of Biological Sciences, São Paulo State University - UNESP, São José do Rio Preto, SP 15054-000, Brazil; f Faculty of Philosophy, Sciences and Letters at Ribeirão Preto - USP, Ribeirão Preto, SP 14040-901, Brazil

**Keywords:** exfoliative toxin C, *Mammaliicoccus sciuri*, cell necrosis, metabolic pathways, metabolomics

## Abstract

Exfoliative toxins (ETs) are glutamyl endopeptidases (GEPs) belonging to the chymotrypsin-like serine protease family (CLSPs), and they play crucial roles in diverse skin diseases. Specifically, exfoliative toxin C (ExhC), expressed by , is an atypical CLSP and has been classified as a moonlighting protein due to its ability to induce necrosis in specific cell lines, inhibit the phagocytic activity of macrophages, and cause skin exfoliation in pigs and mice. The latter function is attributed to the high specificity of ExhC for porcine and murine desmoglein-1, a cadherin that contributes to cell–cell adhesion within the epidermis. Although the amino acid residues responsible for ExhC-induced necrosis have been identified, the specific cellular metabolic pathways leading to cell death remain unclear. Herein, we employed nuclear magnetic resonance (NMR) and mass spectrometry (MS) to explore the metabolic pathways affected by the necrotic activity of ExhC in the BHK-21 cell line. The metabolic profile of cells exposed to subtoxic doses of ExhC revealed significant alterations in oxidative stress protection, energy production, and gene expression pathways. The data demonstrate the potential mechanisms of action of ExhC and highlight that this toxin causes cellular damage, even at low concentrations.

## Graphical abstract

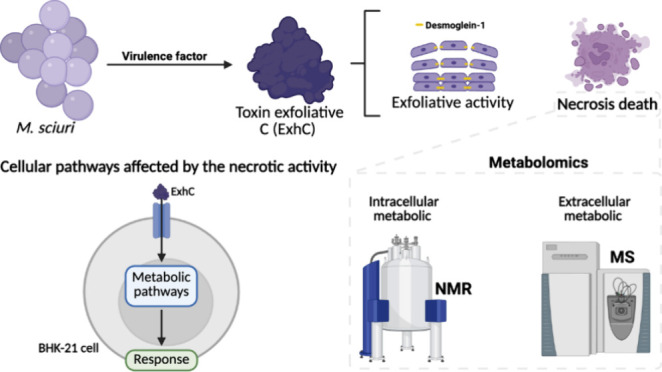

## Introduction

 () is a commensal and pathogenic bacterium of veterinary and clinical relevance.^1^ The species was described as the etiologic agent of exudative epidermitis (EE) in pigs, which causes dehydration and can lead to death within a few days.^2,3^ This skin disease, commonly caused by (), manifests as an acute infection mainly in newly weaned pigs and is characterized by exfoliation accompanied by epidermal cell separation, erythema, and serous exudation.^4^ The main virulence factor in this skin exfoliation is the exfoliative toxin C (ExhC).^3^

ExhC (Figure 1) is a glutamyl endopeptidase belonging to the chymotrypsin-like serine protease family (CLSPs), characterized by the catalytic triad S_195_, H_71_, and D_120_ (Figure 1A).^5,6^ This enzyme is a member of a group collectively known as exfoliative toxins (ETs).^5^ Unlike most members of the chymotrypsin family, ETs are inactive against a wide range of serine protease substrates, showing high specificity for desmoglein-1 (Dsg1), a multidomain transmembrane protein that plays an important role in cell–cell adhesion in the epidermis.^5,7^

**Figure 1:**
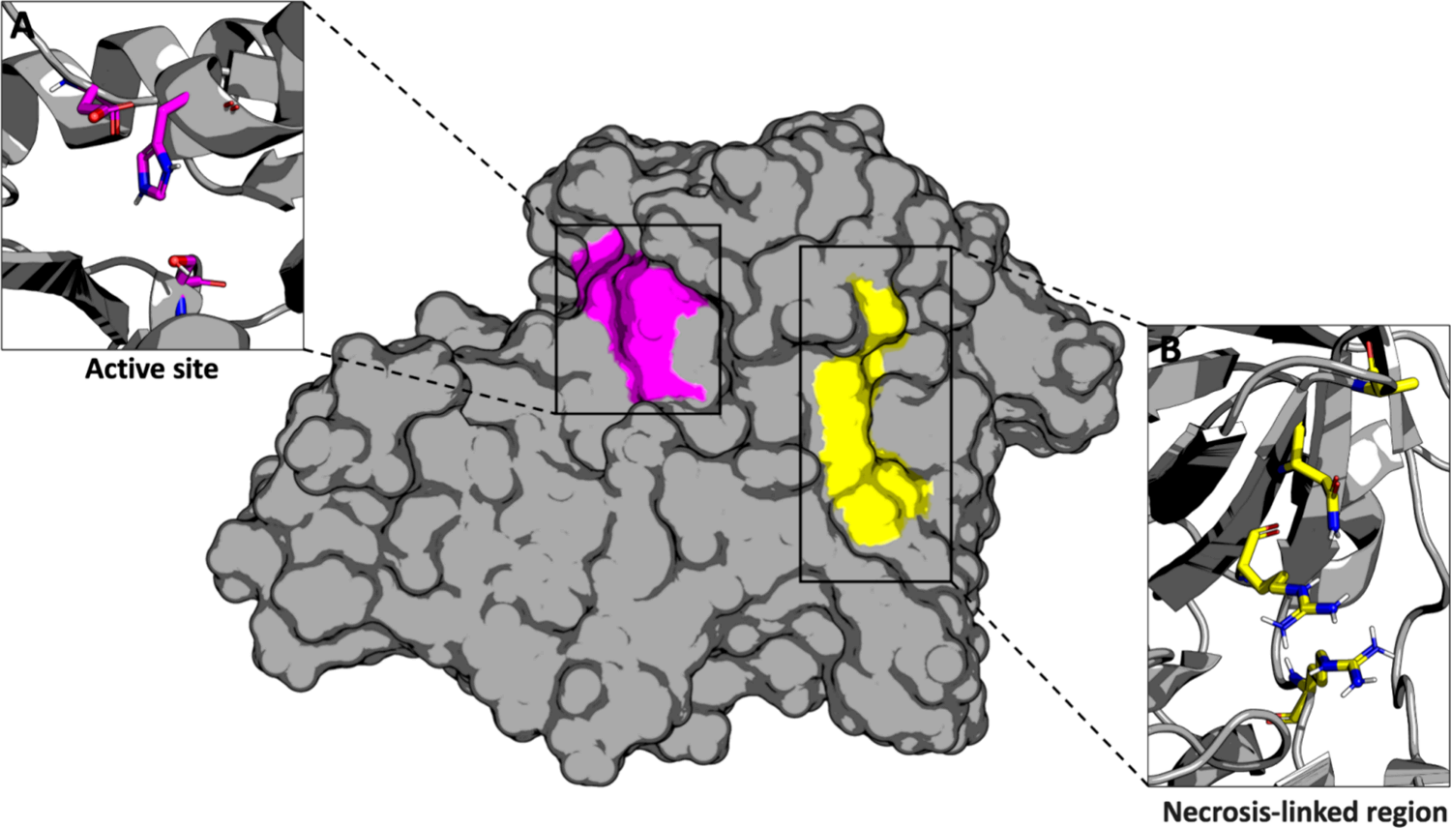
Surface area of the crystallographic structure of exfoliative toxin C (ExhC) from (PDB ID: 8T3J) highlights the cavities containing the residues responsible for the catalytic (magenta) and necrotic (yellow) activities. (A) Amino acid residues S_195_, H_71_, and D_120_ compose the catalytic triad (magenta sticks). (B) Amino acid residues R_47_, N_49_, Q_51_, and R_89_ are involved in necrotic function (yellow sticks).^6^

Apart from causing epidermal exfoliation in pigs and mice, recombinant ExhC from induces necrosis in multiple mammalian cell lines, including renal fibroblasts of newborn hamsters (BHK-21),^8^ and affects the host’s innate immune system by the inhibition of phagocytosis by RAW 264.7 macrophages.^9^ These properties, not observed for any other ET until now, suggest that, in addition to skin exfoliation, ExhC may be involved in other biochemical functions during and infections, especially considering that ExhC expressed by these two species shares an identical amino acid sequence.^8^ Remarkably, the ability to display orthogonal functions is a characteristic found in certain proteins, a phenomenon referred to as moonlighting.^10^

Experiments conducted by Li et al. (2011a) revealed that an ExhC_79–128_ fragment (or an ExhC_44–92_ fragment in the absence of the signal peptide) can trigger necrosis,^8^ and the absence of specific residues R_47_, N_49_, Q_51_, and R_89_ (Figure 1B) abrogates the necrotic activity of ExhC.^6^ Of note, such mutations do not significantly modify the esterolytic activity of the protein, indicating that the necrotic and catalytic activities are independent functions involving distinct protein sites.^6^ The detection of a cavity containing all the residues implicated in necrotic activity (Figure 1B) is an interesting finding that provides evidence of the interaction of ExhC with biomolecules of the cells susceptible to undergoing necrosis.^6^ However, the specific cellular metabolic pathways involved during this process, as well as the process by which cells respond to ExhC during infection, are yet to be elucidated. Herein, the necrosis property of ExhC was evaluated via an *in vitro* assay using BHK-21 cell lines, and the metabolic profiles were analyzed by NMR and MS methods.

## Experimental Section

### Expression and Purification of ExhC

A single bacterial colony of BL21­(DE3) transformed with the pET-28a­(+)-ExhC vector (GenScript) was grown for 16 h at 37 °C in a Lysogeny Broth (LB) medium supplemented with kanamycin (50 mg.mL^–1^).^6^ The culture grown overnight was diluted 100-fold in a fresh LB medium and incubated at 37 °C under vigorous agitation until absorption at 600 nm (A_600_) of 0.5 was attained.^6^ Subsequently, the cells were induced with 0.5 mmol L^–1^ IPTG and incubated at 30 °C for 5 h.^6^ The culture was subsequently centrifuged at 2,600 *xg* for 10 min at 4 °C, and the cells were ruptured by sonication in a lysis buffer, 5 mmol L^–1^ NaHPO_4_, pH 7.7, 400 mmol L^–1^ NaCl, 10 mmol L^–1^ imidazole, and 10% (v/v) glycerol.^11^ The lysed cells were centrifuged at 15,000 *xg* for 30 min at 4 °C, and the pellet obtained was discarded.^6^ The supernatant was subjected to affinity chromatography using an immobilized nickel column (GE) under native conditions and further purified using a Superdex G75 10/300 GL column.^12^ The results were analyzed by 15% SDS-PAGE gel and Western blot.

### BHK-21 Cell Culture

The baby hamster kidney fibroblast cells (BHK-21) (ATCC CCL-10) were cultured in Dulbecco’s modified Eagle’s medium (DMEM, Cultilab, Campinas, SP, Brazil) supplemented with 10% fetal bovine serum (FBS, Gibco—Thermo Fisher Scientific, Waltham, MA, USA), 1% nonessential amino acids (Gibco—Thermo Fisher Scientific, Waltham, MA, USA), and 200 UI.mL^–1^ penicillin and streptomycin (Vitrocell, Campinas, SP, Brazil). The cells were maintained in a humidified incubator at 37 °C in 5% CO_2_.

### Cytotoxicity Assay

The cytotoxicity of ExhC from in BHK-21 was evaluated using a 3-(4,5-dimethylthiazol-2-yl)-2,5-diphenyltetrazolium bromide (MTT) assay as previously described.^6^ The cells (1 × 10^4^ per well) were seeded in 96-well plates for 24 h and incubated with ExhC for 12, 24, 48, and 72 h. Concentrations between 0.94 and 60 μmol L^–1^ protein were tested.^6^ Subsequently, the medium containing the protein was removed, and 1 mg.mL^–1^ MTT (Sigma-Aldrich, St Louis, MO, USA) was diluted into 100 μL of the medium within each well and incubated for 30 min at 37 °C. The medium containing MTT was then removed, and 100 μL of dimethyl sulfoxide—DMSO (Sigma-Aldrich, St. Louis, Missouri, USA) was added. The plate was agitated at 200 rpm. After 5 min, the absorbance was measured at a wavelength of 572 nm on a plate reader (FLUOstar Omega/BMG LABTECH, Ortenberg, Germany). These assays were also performed for the control experiments: 1 × 10^4^ cells with DMEM (i) and 1 × 10^4^ cells with DMEM and PBS 1x (ii).

All experiments were performed in triplicate and evaluated with independent assays. For statistical analysis, the mean ± standard deviation (SD) of the cytotoxicity assays of the BHK-21 cells treated with ExhC was statistically analyzed using GraphPad Prism 8 (GraphPad Software Inc., San Diego, CA, USA). Dunnett’s test was used to compare the control experiment and treated groups. Values of *p* < 0.001 were considered statistically significant.

### Cellular Culture for Metabolite Extraction

Around 1.2 × 10^7^ BHK-21 cells were seeded in 60 mm plates for 24 h and, then, treated with 7.5 μmol L^–1^ ExhC for 48 h. After treatment, the cells were detached with 0.25% trypsin-EDTA (0.25%) (Gibco, Thermo Fisher Scientific, Waltham, MA, USA), followed by two washes with PBS 1x, and the addition of 2 mL of 80% methanol (Sigma-Aldrich). The cell solution was vortexed for 1 min followed by centrifugation at 4000 rcf for 20 min at 4 °C. The aqueous extract obtained was lyophilized in a vacuum centrifuge (Concentrator 5301, Eppendorf, Hamburg, Germany) and stored in the freezer at −80 °C. The extraction of intracellular metabolites was also performed with 1.2 × 10^7^ of BHK-21 cells after 48 h of growth in a DMEM medium, representing the control experiment. Overall, 5 samples of intracellular metabolites were obtained in the presence of ExhC (i), and 10 samples of intracellular metabolites were obtained in the absence of ExhC (ii) (Figure 2). The intracellular samples were diluted in 700 μL of deuterium oxide with 3-(trimethylsilyl)­propionic-2,2,3,3-d_4_ acid sodium salt TSP (D_2_O 99.9%, Sigma-Aldrich) and transferred to 5 mm tubes for data acquisition by nuclear magnetic resonance (NMR) (Figure 2).

**Figure 2:**
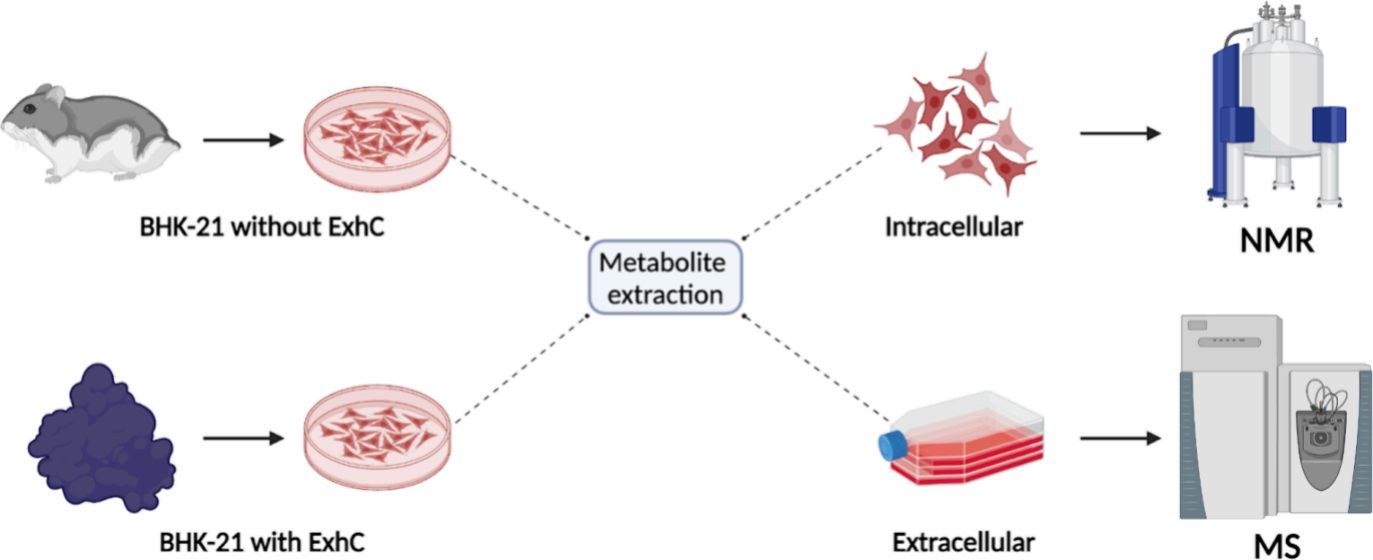
Illustration of the methods used to identify intracellular and extracellular metabolites affected in BHK-21 cells after treatment with ExhC (created with Biorender.com).

In addition, the culture media (extracellular content) were collected from the control and ExhC-treated samples (Figure 2). All samples were filtered by centrifugation (30 min at 4000 rcf) with a 3 kDa membrane (GE Healthcare) and then frozen at −80 °C. Samples were extracted with acetonitrile:water (1:1, v/v), and the organic phase was analyzed by liquid chromatography coupled to tandem mass spectrometry (LC-MS/MS) (Figure 2).

### Nuclear Magnetic Resonance-Based Metabolomics

The 1D and 2D NMR spectra were acquired by using a 600 MHz Bruker Avance spectrometer (Bruker Biospin, Germany) equipped with a triple resonance broadband inverse probe at 25 °C. ^1^H NMR data were obtained using T_2_-edited experiments based on the Carr–Purcell–Meiboom–Gill (CPMG) pulse sequence. This approach enhances the detection of low-molecular-mass metabolites in the sample by filtering broad signals associated with larger molecules.^13^ T_2_-edited ^1^H NMR spectra were acquired using an acquisition time of 3.89 s, a spectral width of 14 ppm, presaturation with a power level of −42.30 dBW, and a 90 ° degree hard pulse of 17.378 W applied for 9.2 μs. Also, presaturation for water suppression was applied. Homonuclear two-dimensional total correlation spectroscopy (TOCSY) was measured using the MLEV pulse sequence with presaturation using 200 scans and the same base parameters, as described. MestreNova (Mnova) software was used for Fourier transformation and phase correction of the spectra. Integration was performed in Mnova for each individual metabolite resonance with the assistance of Chenomx software (Chenomx Inc.). A total of 33 metabolites were quantified.

### Mass Spectrometry-Based Metabolomics

All samples obtained from the extracellular culture media were analyzed by liquid chromatography coupled to a mass spectrometer. The ultrahigh-performance liquid chromatography (UHPLC) system (Nexera X2, Shimadzu-Kyoto, HO, Japan) was equipped with a binary pump system, SIL-30AC autosampler, DGU-20A degasser, and CBM-20A controller. The UHPLC system was interfaced with a TripleTOF5600+ mass spectrometer (Sciex-Foster, CA, USA) equipped with an electrospray ionization source and a quadrupole-time-of-flight analyzer (ESI-QToF). LC-MS/MS data were acquired in untargeted mode. The mobile phases employed were water (phase A) and acetonitrile (phase B), both containing 0.1% formic acid. The chromatographic column employed was an Ascentis C18 (4.6 × 100 mm, 2.8 μm, Supelco), with a flow rate of 0.4 mL.min^–1^. Ten microliters of each sample were injected.

### LC-MS/MS Data Processing

The data were converted to mzML by MSConvert and processed in MZmine v. 2.53.^14^ Signal-to-noise ratios of 1.0E3 and 1.0E1 were considered for MS and MS/MS, respectively, at centroid mode. The ADAP chromatogram builder was employed to reconstruct the chromatogram, considering the min group size in 2 scans, a group intensity threshold of 1.0E3, and an *m*/*z* tolerance of 20 ppm. The local minimum search algorithm (10% as the minimum relative height, 2.0E3 as the minimum absolute height, and 1.2 as the minimum ratio of peak top/edge) was employed for chromatogram deconvolution. Isotopes from the same compound were grouped (*m*/*z* tolerance set at 20 ppm, an RT tolerance of 0.1 min, a maximum charge of 3, and representative isotope set to the most intense). The Join aligner method (an *m*/*z* tolerance of 20 ppm, weight for an *m*/*z* of 75 and an RT of 25, and an RT tolerance of 0.1 min) was used to align the resulting peak list. The resulting table containing the peak areas (.csv) and the MS/MS spectral summary (.mgf) were exported and uploaded in the Global Natural Products Social Molecular Networking Platform (GNPS) (gnps.ucsd.edu).^15^ The quantitative data (.csv) were evaluated by statistical analyses using the MetaboAnalyst platform. A spectral search analysis was performed in GNPS library for compound annotation, using 0.65 as cosine score similarity and at least 4 fragment ions match.

### Chemometric Analysis

Metabolite concentration data, as normalized by TSP, were uploaded in MetaboAnalyst 6.0.^16^ Principal component analysis (PCA) was used to indicate differences in the metabolic profile of control and ExhC-treated cells.^17^ Biplot was used as a graphical representation that combines both PCA loadings (representing the contribution of variables to the principal components) and PCA scores (representing the projection of samples in the reduced-dimensional space), providing a comprehensive view of the relationships between metabolite concentrations and sample groups in the data set.^18^ The direction and length of the vectors represent the contribution and importance of each variable to the principal components, while the proximity of sample points to these vectors provides insights into their metabolic profiles.

### Pathway Analysis

The list of identified metabolites that were relevant for discriminating control and ExhC-treated cells was used in the pathway analysis and enrichment analysis^19^ modules of MetaboAnalyst 6.0 ^16^ to determine the main metabolic pathways affected in BHK-21 cells in the presence of the ExhC protein. The KEGG data set of thewas used for the pathway analysis.^20^

## Results and Discussion

### Cytotoxicity Activity of ExhC in Cell Line BHK-21

Recombinant ExhC was expressed and purified as confirmed by 15% SDS-PAGE gels (Figure 3A). The cytotoxicity assays with ExhC in BHK-21 revealed significant cell death at toxin concentrations of 15, 30, and 60 μmol L^–1^, reducing cell viability by approximately 44, 58, and 64%, respectively, after 48 h of incubation (Figure 3C and Table S1, Supporting Information). In contrast, the addition of ExhC concentrations starting at 7.5 μmol L^–1^ did not cause a significant decrease in the viability of BHK-21 cells (Figure 3C and Table S1, Supporting Information). These results confirm that BHK-21 cell death depends on the toxin concentration, as previously described.^6,8^

**Figure 3:**
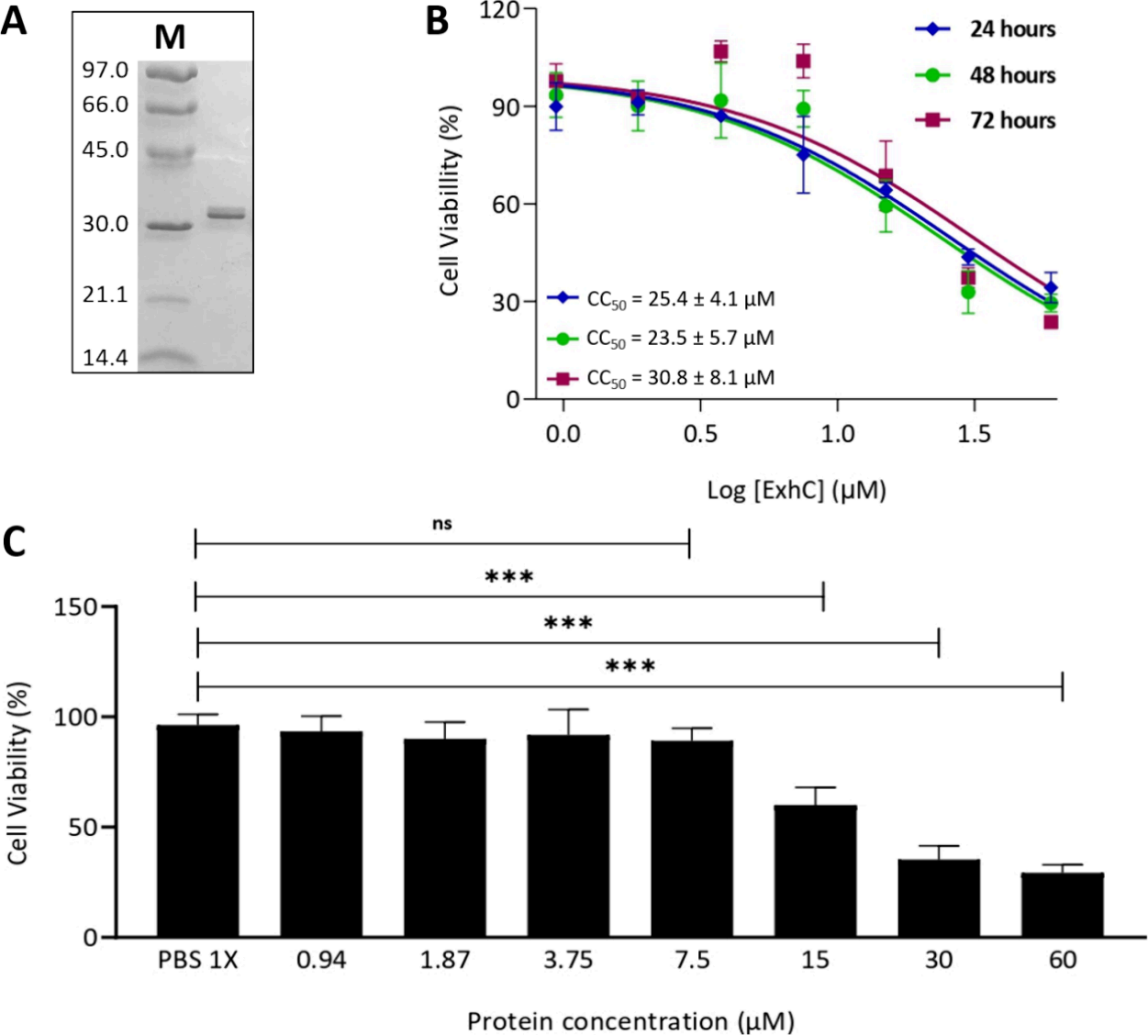
(A) SDS-PAGE of ExhC using the protein molecular weight marker LMW-SDS Marker KIT—GE Healthcare in kDa (M). (B) The dose–response curve represents the 50% cytotoxic concentration (CC_50_) of ExhC in BHK-21 cells following incubation for 24, 48, and 72 h. Triplicate experiments are presented as mean ± SD. (C) The plotted results represent the mean ± SD of viability assays of BHK-21 cells with concentrations between 60 and 0.94 μmol L^–1^ ExhC after 48 h of incubation. Statistically significant differences between the control experiment with PBS 1x and the treated groups were calculated using Dunnett’s test. *** *p* < 0.001; ns, not significant.

Furthermore, half of the cytotoxic concentration (CC_50_) of ExhC was determined to be 25.4 ± 4.1 μmol L^–1^, after 24 h of incubation with BHK-21 (Figure 3B). Interestingly, the dose–response curves after 48 and 72 h of incubation were also generated and indicated CC_50_ values similar to those obtained for a 24 h incubation of ExhC in BHK-21 (Figure 3B). The similarity in CC_50_ values, independent of the increased time-incubation, indicates that ExhC acts constantly but does not prevent the multiplication of still viable BHK-21 cells. The addition of ExhC for 12 h was insufficient to significantly reduce the viability of BHK-21, regardless of the toxin concentration (Figure S1, Supporting Information).

The concentration of 7.5 μmol L^–1^ ExhC incubated for 48 h with BHK-21 cells, which did not cause a significant decrease in cell viability (Figure 3C), was chosen for the potential identification of cellular metabolic pathways affected by the presence of this bacterial toxin. In this sense, BHK-21 cells with ExhC (treated group) and BHK-21 cells without ExhC (control group) were disrupted for the extraction and identification of intracellular metabolites by NMR (Figure 2). The culture media stored from the cultivation of cells from the ExhC-treated and control groups were used to identify extracellular metabolites by MS (Figure 2).

### Intracellular Metabolic Changes Induced by ExhC

Thirty-three metabolites were assigned to the NMR spectra obtained for the treated (Figure 4A) and control (Figure 4B) samples. Among these, 30 were identified with high precision (Table S2, Supporting Information) by comparing the experimental data with the references from the HMDB database^21^ and Chenomx NMR Suite.

**Figure 4:**
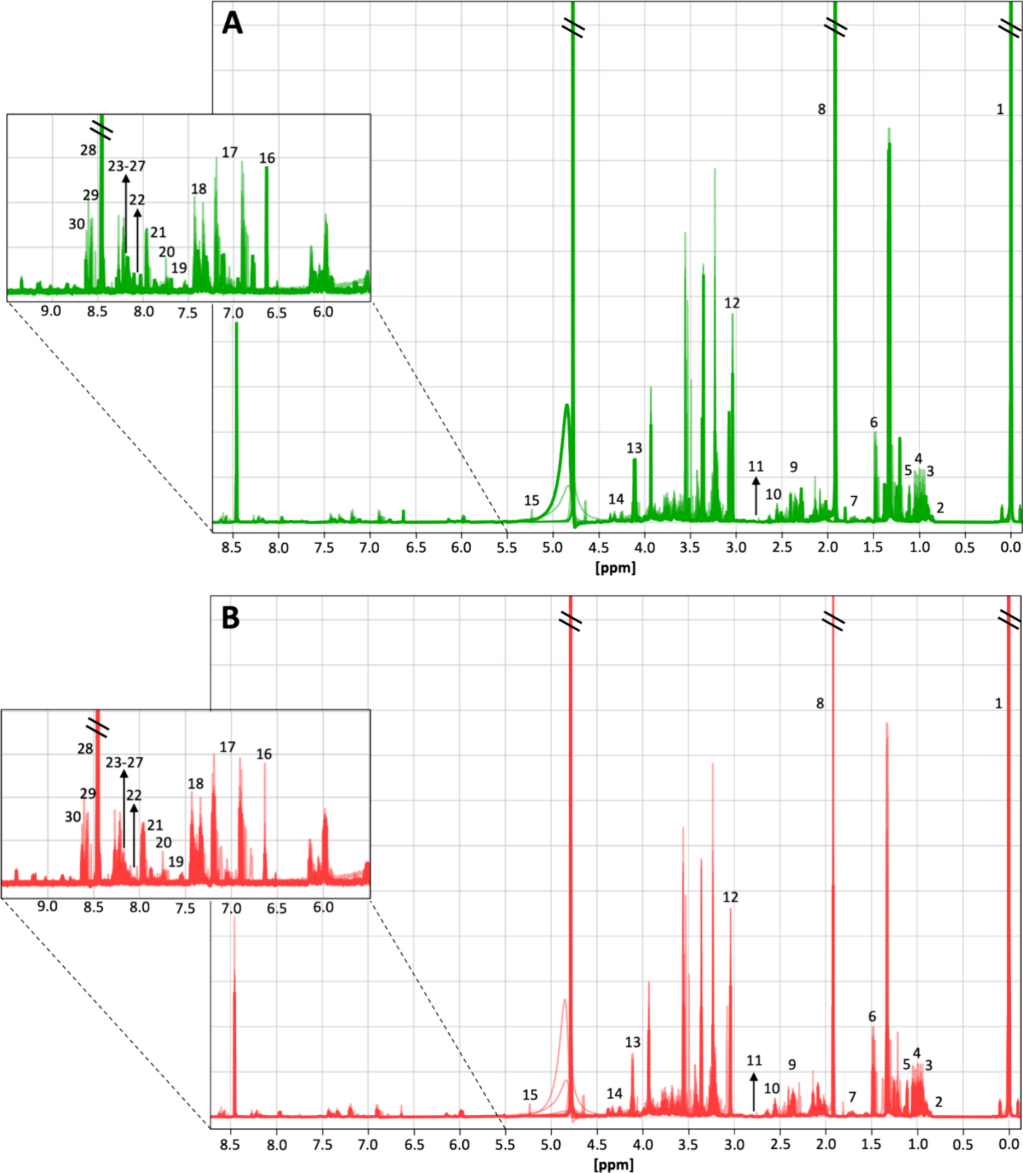
Superposition of ^1^H NMR spectra for the ExhC-treated (A) and control (B) groups. The inset provides a detailed view of the aromatic region of the spectra. Identified metabolites are numbered—1: TSP, 2: valerate, 3: leucine, 4: isoleucine, 5: valine, 6: alanine, 7: butyrate, 8: acetate, 9: succinate, 10: beta-alanine, 11: methylamine, 12: creatine, 13: lactate, 14: proline, 15: glucose galactose, 16: fumarate, 17: tyrosine, 18: phenylalanine, 19: tryptophan, 20: tau-methylhistidine, 21: UDP variants, 22: UMP, 23: GTP, 24: oxypurinol, 25: ATP, 26: NAD^+^, 27: NADP^+^, 28: formate, 29: IMP, and 30: AMP.

To evaluate the impact of the ExhC presence in the BHK-21 cells, PCA obtained by the Euclidean distance metric was applied to the NMR data. The PCA (Figure 5A) evidenced chemical similarity among samples within the same group and a distinct metabolic profile between the control (red) and ExhC-treated (green) cells. A total variance of 92.6% was observed, with PC 1 accounting for 85.5% and PC 2 accounting for 7.1% (Figure 5A). This indicates that the first two principal components effectively capture most of the original variability in the data set. The expressive differentiation between groups in the presence (green) and absence (red) of the toxin by an unsupervised chemometric method (Figure 5A) confirmed that, at subtoxic doses, the ExhC from (Figure 3C) is capable of causing disturbances at metabolic levels in BHK-21 cells.

**Figure 5:**
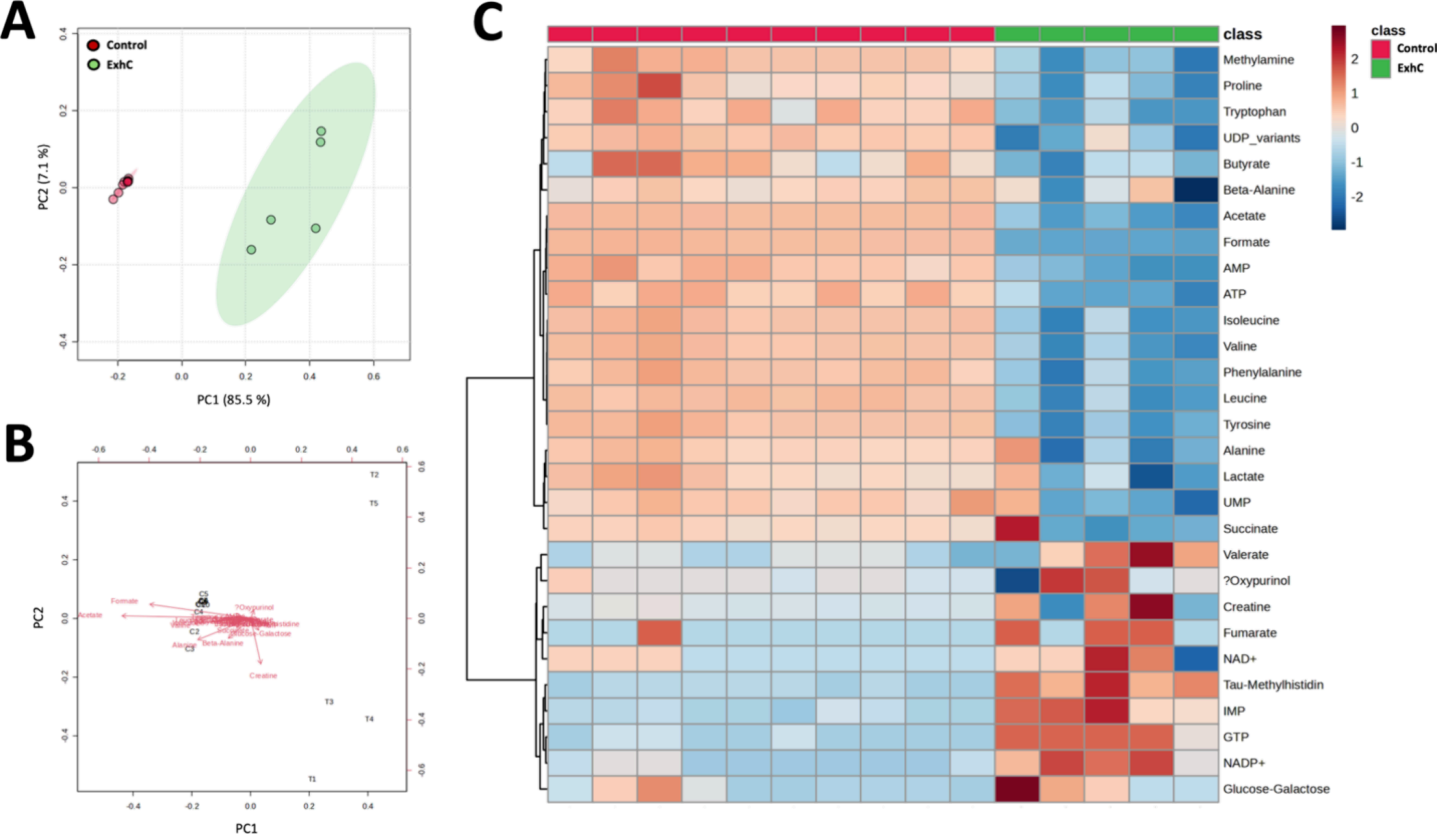
Metabolic profile differences of the BHK-21 cells between the control and ExhC-treated groups. (A) The PCA score plot shows considerable discrimination between the control (red circles) and treated (green circles) groups. (B) PCA biplot (score plots and loadings of variables) from quantified metabolites of the control group and treated group. (C) Hierarchical clustering through heat map analysis using Euclidean distance. The lines represent the discriminant variables from the PCA analysis for the control group (class red) and the ExhC group (class green). The columns indicate the samples, and the color bars on the right depict the relative concentrations of the discriminant metabolites (red: higher relative concentration; blue: lower relative concentration).

Significant differences between the intracellular metabolites of the ExhC-treated and control groups were observed using the PCA biplot (Figure 5B), heat map (Figure 5C), and violin plots (Figure S2, Supporting Information). The decrease or increase in certain metabolites likely occurred in the treated group due to ExhC activity in BHK-21 cells (Figure 3C).

Enrichment analysis (Figure S3, Supporting Information) indicated that glycolysis and pyruvate metabolism were impacted by the addition of ExhC in BHK-21 cells. Complementing these data, reduced levels of pyruvate and acetyl-CoA precursors, such as lactate,^22^ butyrate,^23^ and acetate (Figure 6 and Figure S2, Supporting Information),^24^ suggest an increase in the oxidation of these molecules to feed the tricarboxylic acid (TCA) cycle, allowing the production of cellular energy (Figure 6).^22–25^

**Figure 6:**
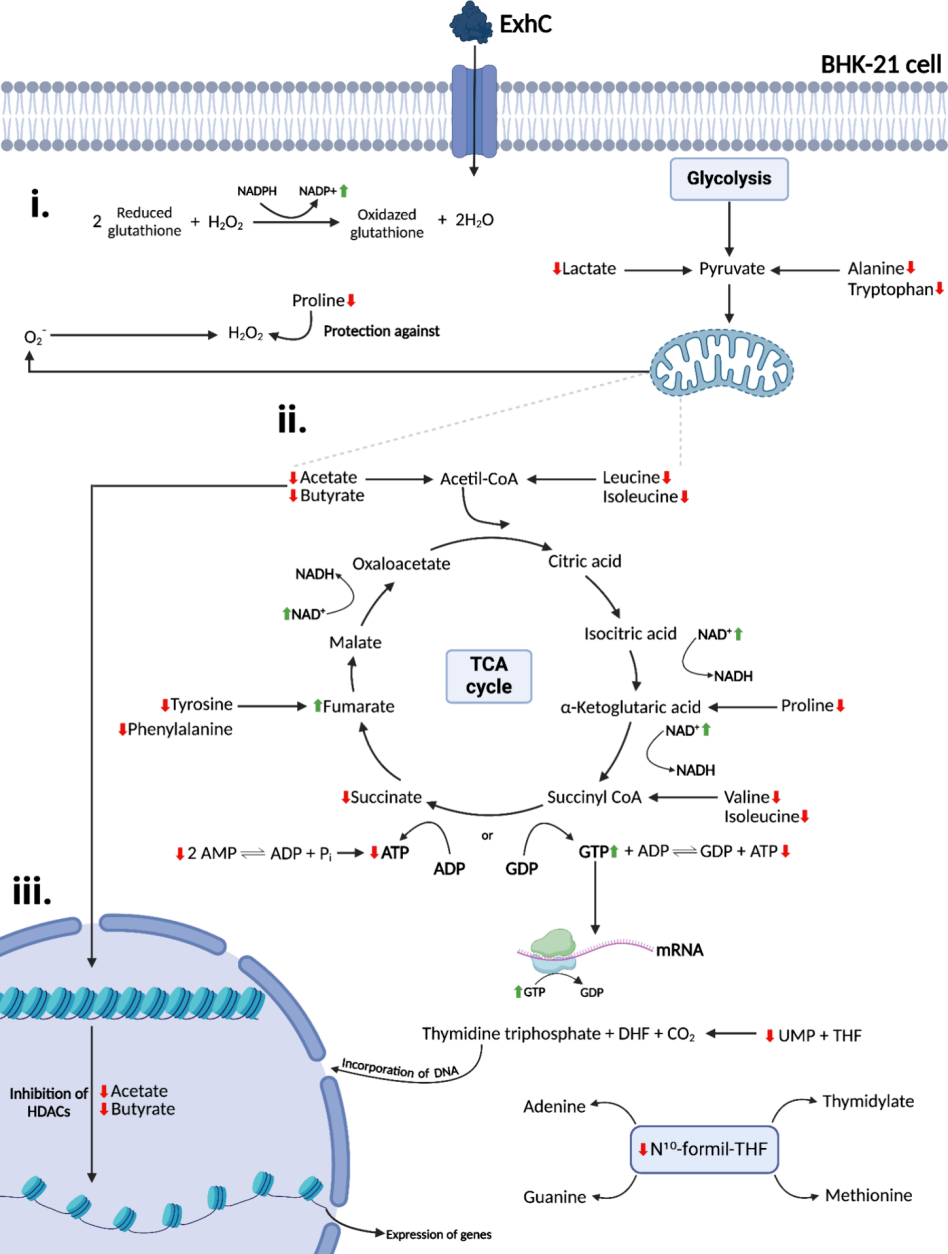
Major pathways were identified as altered in BHK-21 cells treated with ExhC. (i) Mitochondrial ROS production and antioxidant cellular activity. (ii) Tricarboxylic acid (TCA) cycle. (iii) Gene expression. Downward and red (↓) and upward and green (↑) arrows indicate decreased and increased metabolites in ExhC-treated cells compared to the control group, respectively (created with Biorender.com).

This was probably due to the upregulation of the TCA cycle observed in ExhC-treated cells through a decrease in succinate followed by an increase in fumarate (Figure 6 and Figure S2, Supporting Information).^25^ The oxidation of succinate to fumarate is catalyzed by succinate dehydrogenase,^26^ and the data suggest an increase in the activity of this enzyme. There was also a higher consumption of energy molecules (ATP) compared to the control group (Figure 5C). The low ATP levels were accompanied by a decrease in AMP (Figure 5C), a molecule that can be converted into ADP and ATP under conditions of low cellular energy (Figure 6).^27^ In addition, the increase in NAD^+^ in cells in the presence of ExhC (Figure 5C) is also indicative of high energy demand.^28^

Alongside the decrease in ATP, GTP levels increased in ExhC-treated cells (Figure 5C and Figure 6). Depending on intracellular conditions, the TCA cycle can preferentially generate GTP instead of ATP (Figure 6), which can be beneficial depending on the cellular metabolic context.^29^ GTP is crucial for protein synthesis, as it is used by elongation factors to ensure the binding of aminoacyl-tRNAs to the ribosome, as well as for ribosome translocation along the mRNA (Figure 6).^30^

All these variations detected in the activity of the TCA cycle (Figure 6) in ExhC-treated cells explain the ability of this bacterial toxin to cause cell necrosis,^6,8^ considering that energy disturbances are commonly observed in necrotic cells.^31^ Furthermore, since lactate functions as a redox buffer, the decrease in its concentration in treated cells (Figure S2, Supporting Information) likely led to intracellular acidification, resulting in a loss of NAD^+^ regeneration capacity and ATP depletion through the TCA cycle.^22^

Besides being precursors of acetyl-CoA, butyrate and acetate influence the regulation of gene expression, acting directly to inhibit histone deacetylases (HDACs) (Figure 6).^32,33^ The low concentration of these metabolites in cells treated with ExhC (Figure 5C) may have increased the activity of HDACs, ensuring intense chromatin condensation in BHK-21 cells.^33^ This process may have led to a decrease in DNA/RNA replication/transcription and, consequently, inhibition of the translation of proteins crucial for maintaining the viability of the target cells.^33,34^ The low concentration of UMP (Figure 5C) may also have interfered with the synthesis of the genetic material in ExhC-treated cells, as UMP is an essential precursor for the synthesis of pyrimidine nucleotides (Figure 6).^35,36^

Another key metabolite involved in DNA/RNA replication and transcription is formate (Figure S2, Supporting Information), which, along with acetate, is one of the most significant contributors to the differentiation between control and ExhC-treated metabolic profiles (Figure 5B). The significant decrease of formate in BHK-21 cells treated with ExhC may have caused intense inhibition of protein and nucleotide synthesis since the active form of this metabolite (N^10^-formyl-THF) is involved in the synthesis of methionine, purines (adenine and guanine), and thymidylate, a thymine precursor (Figure 6).^37^ Interestingly, enrichment analysis (Figure S3, Supporting Information) indicated that purine and pyrimidine metabolism is affected by ExhC activity.

The concentration of the amino acids proline, tryptophan, isoleucine, leucine, valine, phenylalanine, tyrosine, and alanine also decreased in ExhC-treated cells compared to the control group (Figure 5C and Figure S2, Supporting Information). In this sense, the metabolic pathways of these residues were affected in the presence of ExhC (Figure S3, Supporting Information). It is important to note that these amino acids are not only crucial for protein synthesis but also capable of interacting with other metabolic pathways.

Proline, for example, can modulate the intracellular redox environment and protect mammalian cells against oxidative stress, specifically from hydrogen peroxide (H_2_O_2_) (Figure 6).^38^ Glutathione, composed of amino acids, also plays a crucial role as an antioxidant, neutralizing H_2_O_2_ (Figure 6).^39^ The effect of ExhC on glutathione metabolism according to enrichment analysis (Figure S3, Supporting Information), along with the increase in the NADP^+^ concentration in ExhC-treated cells (Figure 5C), suggests an upregulation of the antioxidation pathway coordinated by glutathione (Figure 6).^39^ Altogether, these data indicate the presence of oxidative stress in cells incubated with ExhC.

Oxidative stress is another biological condition that can cause cell necrosis,^40^ and the postulated upregulation of the TCA cycle in ExhC-cells would intensify the production of this reactive oxygen species (ROS) by mitochondria (Figure 6).^41^ Additionally, all of the amino acids indicated here can be used indirectly to upregulate the TCA cycle (Figure 6), and their reduced concentrations are consistent with the hypothesis of energy disturbance in BHK-21 cells in the presence of ExhC.

### Extracellular Metabolic Changes Induced by ExhC

MS analyses identified significant differences in the concentrations of four extracellular metabolites when comparing the control and ExhC-treated cells (Figure 7). These compounds are level 2 of annotation according to the Metabolomics Standards Initiative (MSI) (Figure S4, Supporting Information).^42^ Two of these metabolites are common structural components (Figure S5, Supporting Information) of the plasma membrane of mammalian cells: 16:0 Lyso-PC (i) and octadecenoyl-glycero-phosphocoline (ii).^43,44^ The increased concentration of these phospholipids in the extracellular environment of the ExhC-treated group (Figure 7) is indicative of damage and/or rupture of the plasma membrane of BHK-21 cells.^45^ The loss of membrane integrity is characteristic of cells that suffer abrupt death,^31^ which is again in line with the data showing ExhC as a toxin capable of causing necrosis in mammalian cells.^6,8^

**Figure 7:**
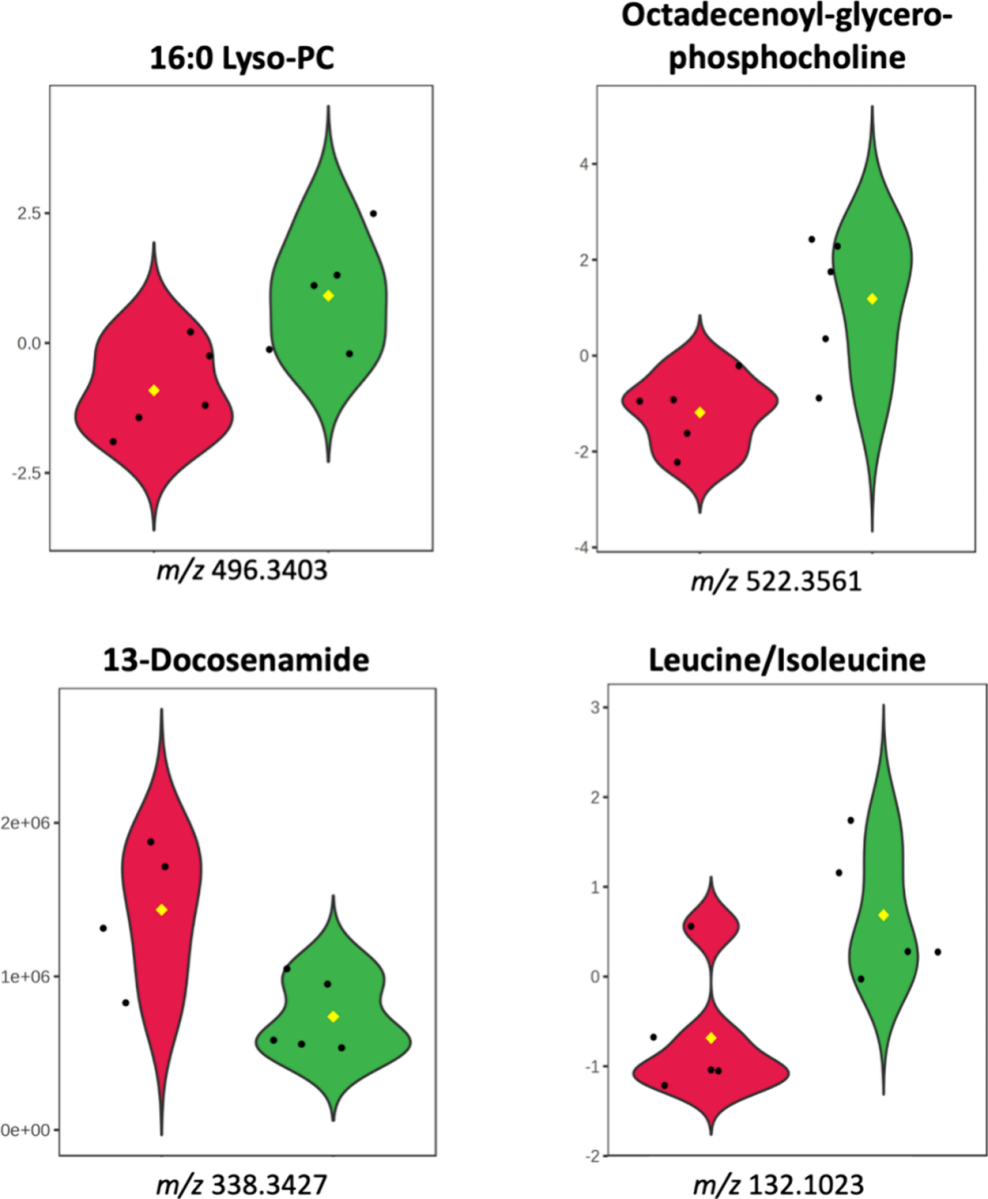
Violin plots of extracellular metabolites with significant statistical differences between the ExhC-treated cells and controls. These metabolites were annotated by spectral library search (level 2).^42^ The black points represent the samples, and the colored area represents the distribution of the samples, ExhC-treated cells (green) and controls (red). *t*-test (*p*-value < 0.05) of metabolites highlighted by the methods employed.

The decrease in 13-docosenamide (or erucamide) (Figure S5, Supporting Information) levels was also an interesting artifact detected in the extracellular environment of ExhC-treated cells (Figure 7). Interestingly, this fatty acid amide is described as a component that causes high angiogenic activity in different mammals,^46^ being responsible for stimulating the formation of blood vessels, as well as modulating the extracellular water balance.^46,47^

In addition, the increased concentration of leucine/isoleucine (Figure S5, Supporting Information) in the extracellular environment of ExhC-treated cells (Figure 7) corroborates the low levels of these amino acids in the intracellular environment (Figure 5C and Figure S2, Supporting Information). These data indicate that the intracellular decrease in leucine/isoleucine occurred because it was directed to the environment outside the cell via the plasma membrane. It is important to note that, when in an environment external to the cell, isoleucine/leucine can act on cell signaling pathways, influencing processes of proliferation, differentiation, and response to stress through activation via mTOR.^48–50^

## Conclusions

This study evaluated the metabolic profiles of BHK-21 cells in the absence and presence of exfoliative toxin C (ExhC) from , which in certain concentrations is capable of causing necrosis in different mammalian cell lines. To date, ExhC is the only exfoliative toxin which, in addition to causing exfoliation of the epidermis, is also capable of inducing cell death. Based on the experimental evidence presented, the concentration of 7.5 μmol L^–1^ ExhC for 48 h did not cause high cytotoxicity in BHK-21 cells; however, it was able to induce significant metabolic changes that indicated the potential pathways by which ExhC may be acting during the process by which this toxin triggers cell necrosis.

The intensification of the TCA cycle, which is indicative of an energy disturbance, accompanied by a decrease in gene expression and alterations in responses to oxidative stress are characteristic events of cell necrosis, and all of them were identified as potentially affected pathways from the analyses of the metabolic profile of the ExhC-cells. In this same group, significant concentrations of phospholipids were observed in the extracellular environment, indicating a loss of plasma membrane integrity, which commonly occurs during the process of abrupt cell death. Overall, the data obtained herein provided information that brings us closer to understanding the mechanistic pathways involved in the necrotic activity of the studied toxin ExhC. However, the cellular targets that interact directly with this toxin during the cell death process still need to be discovered.

## Supplementary Material



## Data Availability

The HPLC-MS and NMR data have been deposited in MassIVE (massive.ucsd.edu) and the Metabolomics Workbench (www.metabolomicsworkbench.org) repositories, under data set IDs MSV000096527 and 5411, respectively.

## Notes

(Table S1) Effects of the progressively increasing concentration of ExhC on the viability of the cell line BHK-21 by cytotoxicity assays after 12, 24, 48, and 72 h of incubation, results were presented as mean ± SD; (Table S2) differential metabolites detected by NMR spectra in ExhC-treated cells compared with controls; (Figure S1) (A) the plotted results represent the mean ± SD of viability assays of BHK-21 cells with concentrations between 60 and 15 μM of ExhC after 12 h of incubation, and the statistically significant difference between the control experiment with PBS 1X and the treated groups were calculated using Dunnett’s test, ns, not significant; (B) dose–response curve of ExhC in BHK-21 cells after incubation for 12 h, and triplicate experiments are presented as mean ± SD; (Figure S2) violin plots of metabolites highlighted in the statistical analysis of intracellular content of BHK-21 cells compared with the cells treated with the ExhC, these metabolites are identified by NMR technique, the black points represent the samples and colored area represents the distribution of the samples, in which the red represent samples from the controls and green represent the treated samples, and *t* test (*p*-value < 0.05) of metabolites highlighted by the methods employed; (Figure S3) summary of pathway enrichment analysis performed in MetaboAnalyst using the set of metabolites indicated as significantly altered in BHK-21 cells with the addition of ExhC compared to the control experiment; (Figure S4) mirror matches spectra of compounds detected by mass spectrometry, these compounds were annotated based on spectral search against the GNPS spectral library, considering cosine similarity score of 0.65 and at least 4 fragment ions match, and this annotation is level 2 according to the Metabolomics Standards Initiative (MSI);^42^ and (Figure S5) structure of compounds identified by mass spectrometry (PDF)
