# Supplementary material for: Metabolic Effects of Cellular Necrosis Caused by Exfoliative Toxin C (ExhC) from
Source: J Proteome Res. 2025 May 22;24(7):3261–71. doi: 10.1021/acs.jproteome.4c01029 (PMC12235709; doi:10.1021/acs.jproteome.4c01029)
Supplement: Supplementary file 1 [file pr4c01029_si_001.pdf]

## SUPPORTING INFORMATION

# Metabolic effects of cellular necrosis caused by exfoliative toxin C (ExhC) from *Mammaliicoccus sciuri*

**Authors:** Carolina Gismene[a,*], Fábio Rogério de Moraes[a], Anelize Bauermeister[a,b], Thyerre Santana Da Costa[c], Marília de Freitas Calmon[d], Luís Eduardo de Almeida Passos Cerbino[a], Paula Rahal[d], Rejane Maira Góes[e], Luiz Alberto Beraldo de Moraes[f], Ljubica Tasic[c], Raghuvir Krishnaswamy Arni[a]

[a] Multiuser Center for Biomolecular Innovation, São Paulo State University - UNESP, São José do Rio Preto, SP 15054-000, Brazil.

[b] Department of Chemistry, Institute of Chemistry, University of São Paulo - USP, São Paulo, SP 05508-000, Brazil.

[c] Institute of Chemistry, Universidade Estadual de Campinas - UNICAMP, Campinas, SP 13083-970, Brazil.

[d] Laboratory of Genomic Studies, São Paulo State University - UNESP, São José do Rio Preto, SP 15054-000, Brazil.

[e] Department of Biological Sciences, São Paulo State University - UNESP, São José do Rio Preto, SP 15054-000, Brazil.

[f] Faculty of Philosophy, Sciences and Letters at Ribeirão Preto - USP, Ribeirão Preto, SP 14040-901, Brazil.

* Correspondence: carolina.gismene@unesp.br

**Table S1.** Effects of progressively increasing concentration of ExhC on the viability of the cell line BHK-21 by cytotoxicity assays after 12, 24, 48, and 72 h of incubation. ....................................................................................................................**2**

**Figure S1.** (A) The plotted results represent the mean ± SD of viability assays of BHK-21 cells with concentrations between 60-15 µM of ExhC after 12 hours of incubation. The statistically significant difference between the control experiment with PBS 1X and the treated groups were calculated using Dunnett's test. ns, not significant. (B) Dose-response curve of ExhC in BHK-21 cells after incubation for 12 hours. Triplicate experiments are presented as mean ± SD..........................................**2**

**Table S2.** Differential metabolites detected by NMR spectra in ExhC-treated cells compared with controls.........................**3**

**Figure S2.** Violin plots of metabolites highlighted in the statistical analysis of intracellular content of BHK-21 cells compared with the cells treated with the ExhC……………………………………………………………………………………**4**

**Figure S3.** Summary of pathway enrichment analysis performed in MetaboAnalyst using the set of metabolites indicated as significantly altered in BHK-21 cells with the addition of ExhC compared to the control experiment. ..................................**5**

**Figure S4.** Mirror matches spectra of compounds detected by mass spectrometry. ..............................................................**6**

**Figure S5.** Structure of compounds identified by mass spectrometry.....................................................................................**6**

**Table S1.** Effects of progressively increasing concentration of ExhC on the viability of the cell line BHK-21 by cytotoxicity assays after 12, 24, 48, and 72 h of incubation. Results were presented as mean ± SD.

| Cell Viability (%) | | | | | | | |
|---|---|---|---|---|---|---|---|
| Concentration (μmol.L$^{-1}$) | 60 | 30 | 15 | 7.5 | 3.75 | 1.87 | 0.94 |
| 12 h | 87.3 ± 7.5 | 89.9 ± 3.9 | 93.0 ± 7.1 | 92.2 ± 4.4 | 93.6 ± 1.7 | 98.5 ± 2.2 | 101.2 ± 4.0 |
| 24 h | 34.3 ± 4.7 | 43.7 ± 2.4 | 64.2 ± 2.4 | 80.1 ± 11.8 | 87.0 ± 1.5 | 91.2 ± 3.9 | 89.9 ± 7.3 |
| 48 h | 36.1 ± 3.8 | 41.8 ± 3.9 | 55.8 ± 10.2 | 89.2 ± 5.6 | 91.8 ± 11.5 | 90.0 ±7.7 | 93.4 ± 7.0 |
| 72 h | 23.0 ± 1.2 | 37.3 ± 3.1 | 68.7 ± 10.7 | 118.9 ± 3.8 | 106.8 ± 3.3 | 92.0 ± 1.9 | 97.8 ± 5.2 |

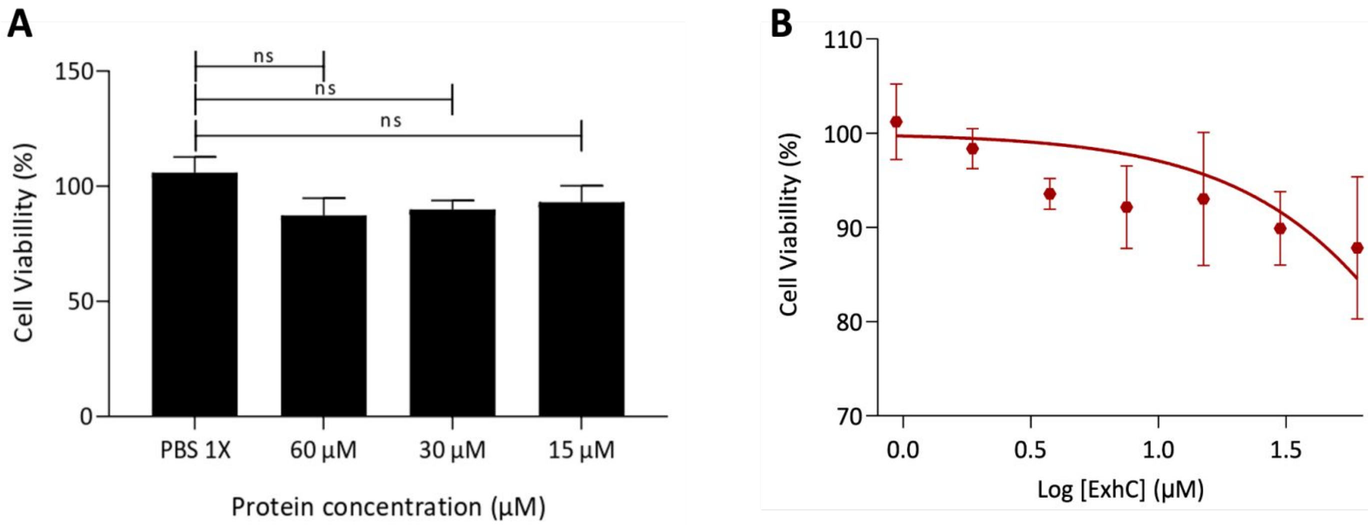


**Figure S1.** (A) The plotted results represent the mean ± SD of viability assays of BHK-21 cells with concentrations between 60-15 μM of ExhC after 12 hours of incubation. The statistically significant difference between the control experiment with PBS 1X and the treated groups were calculated using Dunnett's test. ns, not significant. (B) Dose-response curve of ExhC in BHK-21 cells after incubation for 12 hours. Triplicate experiments are presented as mean ± SD.

**Table S2.** Differential metabolites detected by NMR spectra in ExhC-treated cells compared with controls.

| NMR signals used for quantification (ppm) | Metabolites | ExhC-treated cells | Control cells |
|---|---|---|---|
| 0.885 | Valerate | ↑ | ↓ |
| 7.699 | Tau-Methylhistidin | ↑ | ↓ |
| 6.520 | Fumarate | ↑ | ↓ |
| 3.039 | Creatine | ↑ | ↓ |
| 5.234 | Glucose-Galactose | ↑ | ↓ |
| 8.572 | IMP | ↑ | ↓ |
| 8.445 | NADP+ | ↑ | ↓ |
| 8.440 | NAD+ | ↑ | ↓ |
| 8.153 | GTP | ↑ | ↓ |
| 8.227 | Oxypurinol | ↑ | ↓ |
| 1.569 | Butyrate | ↓ | ↑ |
| 7.958 | UDP-variants | ↓ | ↑ |
| 4.144 | Proline | ↓ | ↑ |
| 2.633 | Methylamine | ↓ | ↑ |
| 1.919 | Acetate | ↓ | ↑ |
| 8.458 | Formate | ↓ | ↑ |
| 8.130 | UMP | ↓ | ↑ |
| 2.552 | Beta-Alanine | ↓ | ↑ |
| 1.017 | Isoleucine | ↓ | ↑ |
| 0.966 | Leucine | ↓ | ↑ |
| 7.188 | Tyrosine | ↓ | ↑ |
| 7.425 | Phenylalanine | ↓ | ↑ |
| 1.043 | Valine | ↓ | ↑ |
| 7.542 | Tryptophan | ↓ | ↑ |
| 8.626 | AMP | ↓ | ↑ |
| 8.270 | ATP | ↓ | ↑ |
| 2.405 | Succinate | ↓ | ↑ |
| 1.474 | Alanine | ↓ | ↑ |
| 1.320 | Lactate | ↓ | ↑ |

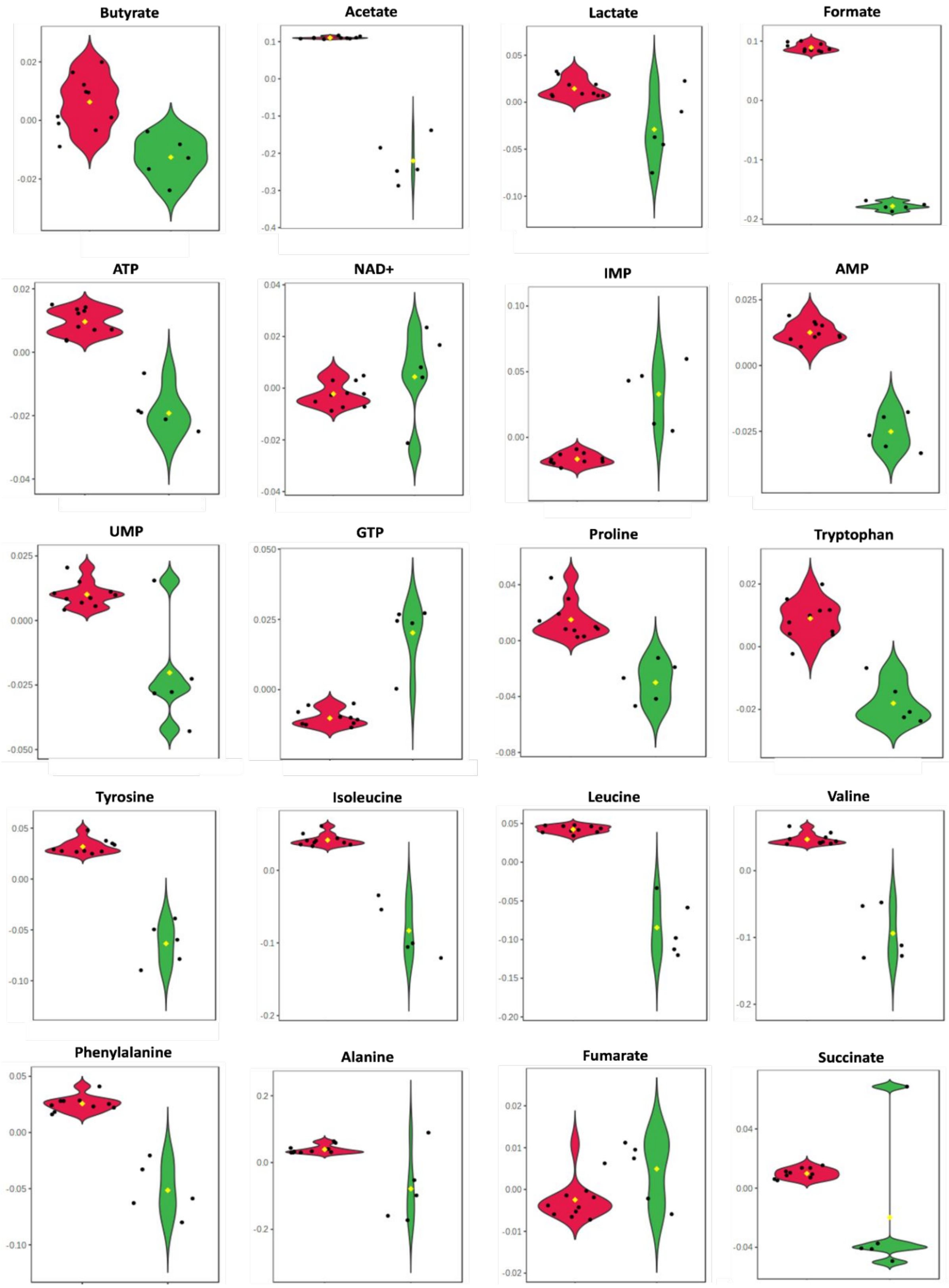


**Figure S2.** Violin plots of metabolites highlighted in the statistical analysis of intracellular content of BHK-21 cells compared with the cells treated with the ExhC. These metabolites are identified by NMR technique. The black points represent the samples and the colored area represents the distribution of the samples, in which, the red represent samples from the controls and the green represent the treated samples. *t*-test (*p*-value < 0,05) of metabolites highlighted by the methods employed.

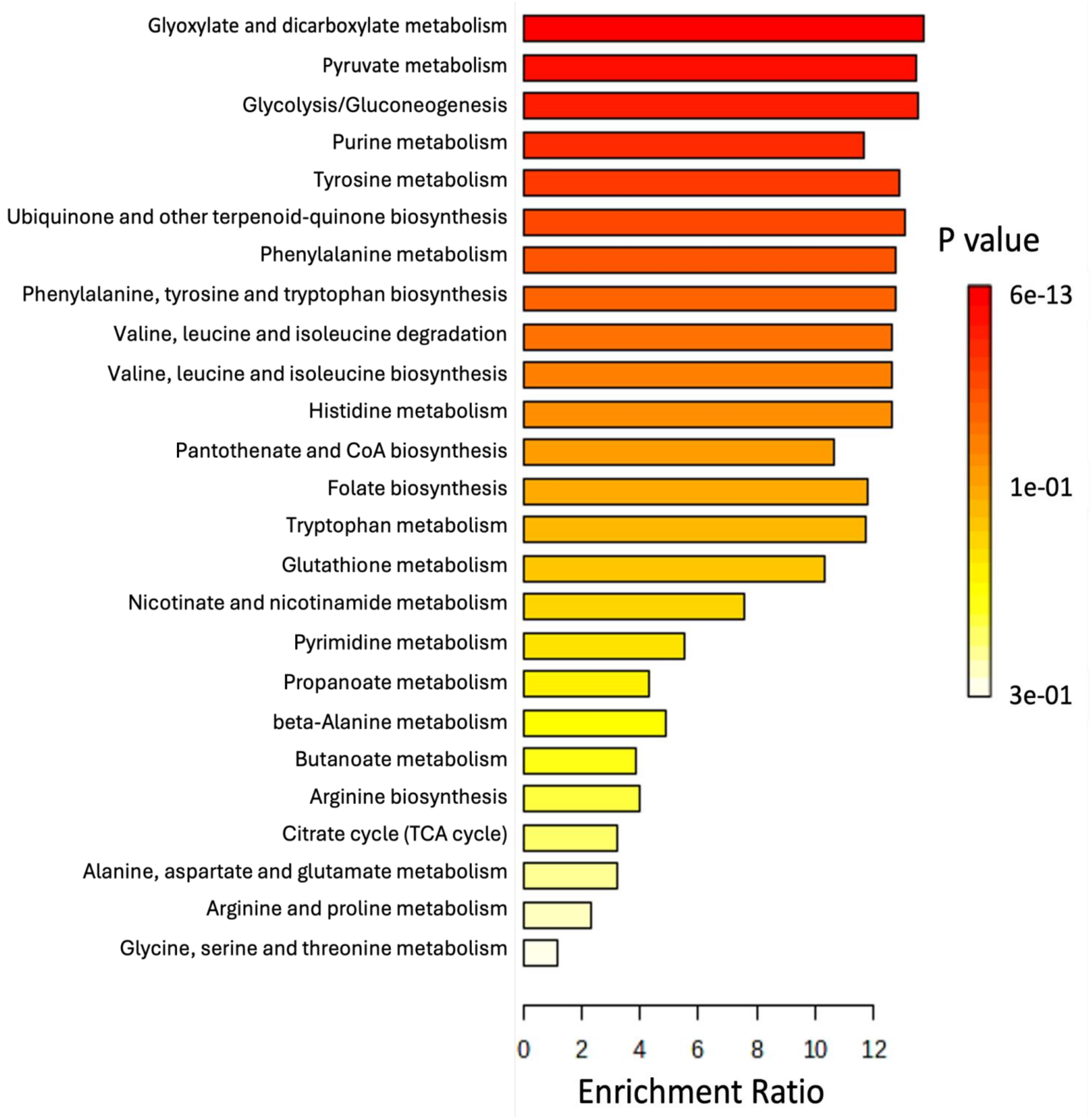


**Figure S3.** Summary of pathway enrichment analysis performed in MetaboAnalyst using the set of metabolites indicated as significantly altered in BHK-21 cells with the addition of ExhC compared to the control experiment.

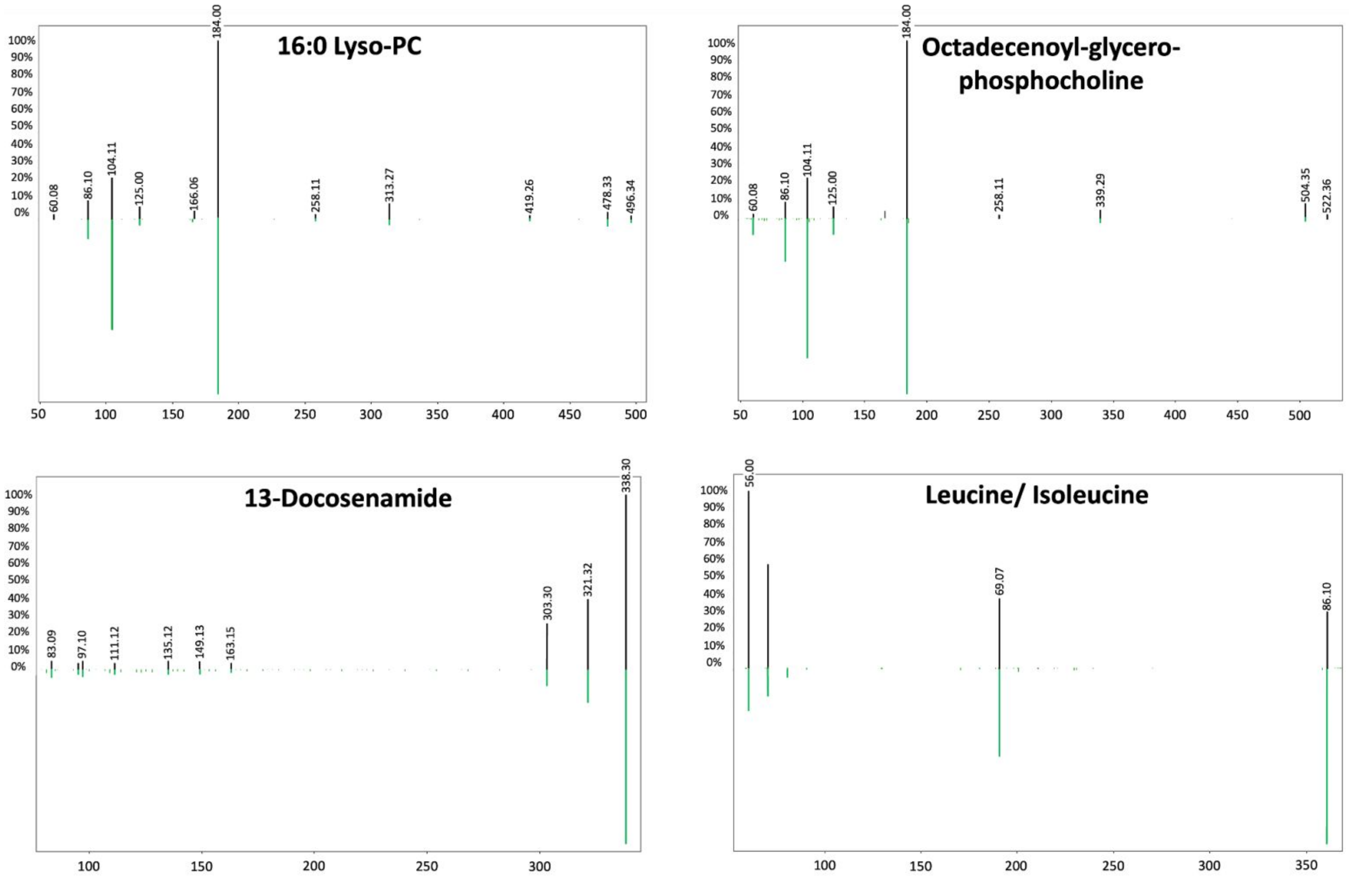


**Figure S4.** Mirror matches spectra of compounds detected by mass spectrometry. These compounds were annotated based on spectral search against the GNPS spectral library, considering cosine similarity score of 0.65 and at least 4 fragment ions match. This annotation is level 2 according to the Metabolomics Standards Initiative (MSI).[42]

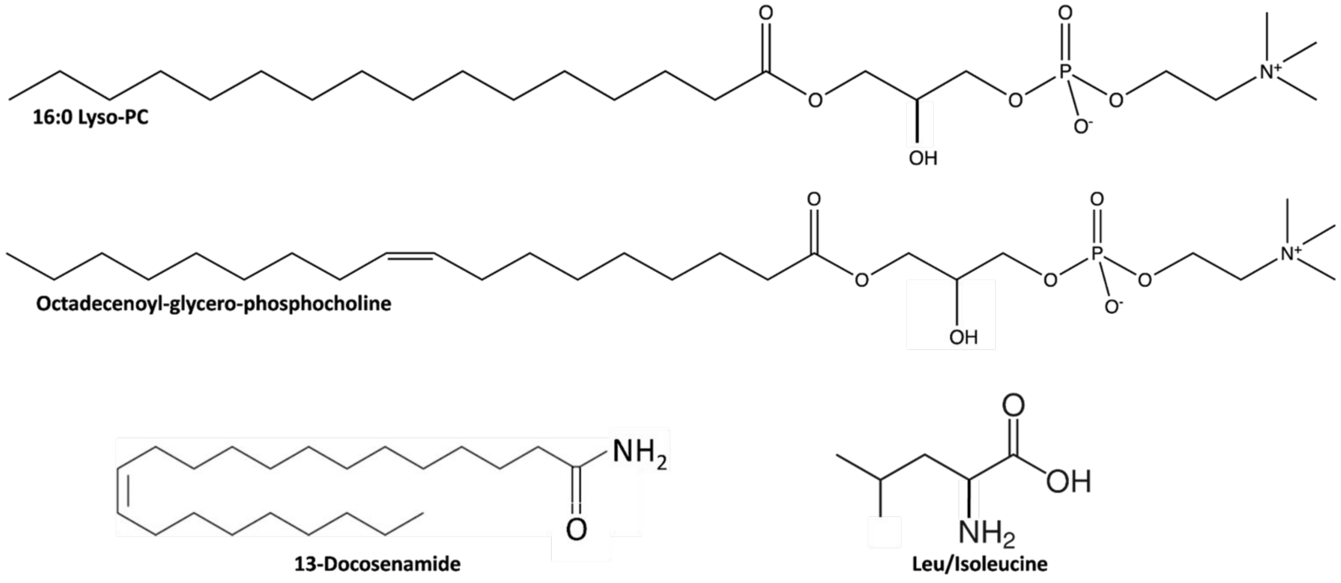


**Figure S5.** Structure of compounds identified by mass spectrometry.
